# A Case of Singular Disease of the Cranium

**Published:** 1819-04

**Authors:** Wm. M'Turk


*For the London Medical and Physical Journal.*

### *A Case of singular Disease of the Cranium;* by Wm. M'Turk, Esq.

IN the spring of 1817, a gentleman began to feel pains in his head, more particularly the back part of it, which were supposed to be connected with the digestive functions, then considerably impaired. He took some medicines, which restored the tone of the stomach, but had no effect in relieving the head. For some months following he did not take any medicine; the pain varying in degree, but nearly incessant. In the summer the symptoms became aggravated, his sight and hearing being considerably impaired. His professional duties were of a sedentary nature, which, with some domestic circumstances at that time operating considerably on his mind, induced him to consider his complaints *nervous*: he, therefore, determined to make trial of Peruvian bark; which was taken for some time without benefit. The pain now became very distressing, yet varied much: sometimes he was nearly free from it, at others it was most intense and his sight nearly lost. He always found that any jolting of the body, in proportion to its violence, increased the pain; so that he avoided, as much as possible, walking on rough uneven roads or down declivities, and always preserved an even pace, carefully guarding against tripping in his progress. In the month of December he had fifteen ounces of blood taken from the back of his neck by cupping, but from which he obtained no mitigation of his symptoms. Since that time up to the beginning of February 1818, when he consulted me, I believe he had not been making use of any means for relief. From some circumstances connected with his history prior to these dates, it was judged advisable that he should take the pilula hydrargyri with decoctum sarsaparillæ; but, at his own request, this was postponed till nearer the spring, from a persuasion that he had that, that season was more favourable for taking medicine. In a few weeks afterwards, however, he requested I would examine his head. I did so, and perceived on the scalp, at equal distances from the coronal and lambdoidal sutures, and directly over the sagittal suture, a tumor about the size of an egg, evidently containing matter. To this a poultice was applied, and in a day or two a small orifice was made, from which issued a considerable discharge of pus, of thick consistence, and peculiarly shining and pearly appearance. He experienced much relief; and in the morning, when I visited him, he was quite free from pain, and had slept the whole night. A large quantity of matter had been evacuated. I proceeded to dress the place; but was greatly surprised by observing a fragment of cerebral matter, larger than a hazel-nut, accompanied by the pia mater, plugging up the orifice which the lancet had made. In the evening of the same day, Dr. Thompson met me in consultation, when the part was examined through a microscope. The ramification of the vessels on the membrane could be most distinctly traced, and the structure of the portion of brain clearly distinguished. It was also discovered that the sagittal suture, and the coronal partially, had given way, and that, by pressure with the hands on each side the parietal bones (separated apparently about the third of an inch), they could be made to approach and recede, as the force was applied; and, by the application of pressure in this way, it was most satisfactorily demonstrated that the matter was forced out from within the skull. It may not be improper to remark also, that the cranium appeared considerably misshaped, very closely resembling the head of a hydrocephalic child. The serrated edges of the bones could be very distinctly felt with the probe. The orifice was cautiously enlarged, and the matter which was effused under the scalp, and had denuded the bone, was gently pressed out. Just below the coronal suture, a little to the right side, another fluctuating tumor appeared, smaller than the former, and which had no communication with it, external to the skull, that could be traced with the probe, yet pressure upon this caused matter to exude abundantly from the upper one.

Not to be tedious in the detail of symptoms which varied but little, it will be sufficient to state that the discharge continued in great quantity for eight or ten days; from which time it gradually lessened, the bones regaining their natural situation, the incisions granulating; and, in three weeks from the opening of the tumors, the patient was perfectly well, with the restoration both of his sight and hearing.

I shall make no comments, well aware how very unsatisfactory must be all attempts to account for this extraordinary, and perhaps unique, case. I can only express my astonishment that disease so extensive in an organ, whose functions are considered so susceptible of derangement and are so important in the animal economy, should not have produced insensibility and death. I have purposely abstained from saying any thing of the treatment, believing that it could add nothing to the value of the communication, as it consisted merely in what the passing symptoms obviously suggested. I may add that, a few months after the date of his recovery, he took, for a stomachic affection, about a dozen of the pilulæ hydrarg. They produced an unpleasant effect; the cicatrices of the wounds becoming the seat of considerable irritation, which terminated in a small superficial formation of matter; and, the pills being desisted from, no further mischief ensued. Since that time he has taken no medicine whatever; and I am happy to say he has enjoyed an invariably good state of health up to the present time, being nearly a year since the appearance of the swelling on his head.

*Scarborough; Feb.* 6, 1819.

